# Assessing the Verbal Behavior of a Linguistically Diverse Speaker with Autism

**DOI:** 10.1007/s40616-023-00196-x

**Published:** 2023-11-20

**Authors:** Sreeja Atherkode, Lee Mason

**Affiliations:** 1 Department of Teacher Education and Administration, University of North Texas, Denton, TX USA; 2 Child Study Center, Cook Children’s Health Care System, 1300 W Lancaster Ave, Fort Worth, TX 76110 USA; 3 Burnett School of Medicine, Texas Christian University, Fort Worth, TX USA

**Keywords:** Autism spectrum disorder, Functional analysis, Heritage language, Multilingualism, Verbal behavior

## Abstract

For speakers belonging to multiple verbal communities, functional analyses of verbal behavior allow for dynamic control over response topography. The simple practice of allowing the speaker the freedom to select the language of instruction minimizes cultural bias and hegemony. We extended the research on functional analyses of verbal behavior to include a speaker of multiple languages in a quasi-experimental case study. We employed verbal operant experimental (VOX) analyses as a repeated measure of language acquisition with a linguistically diverse, 7-year-old Indian boy with autism. The VOX analyses were conducted as part of the child’s early intensive behavioral intervention, and we observed the impact of an immersive foreign language experience on his verbal repertoire with follow-up VOX analyses conducted in three topographically distinct languages: English, Telugu, and Tamil. The results show a dynamic hierarchy of strength between the three languages, with overarching patterns across the three assessments. The implications for using VOX analyses to assess the functional language skills of multilingual speakers with autism are discussed, and areas of future research are highlighted.

Behavior analysts who study verbal behavior are uniquely situated to facilitate the acquisition of foreign-language skills. Approximately 20% of American children are raised bilingual (Wang et al., 2018), while the number of individuals speaking a heritage language – a primary language other than English – tripled between 1980 and 2019 (Dietrich & Hernandez, 2022). Although commercially available verbal-behavior assessments such as the *Verbal Behavior Milestones Assessment and Placement Program* (Sundberg, 2014) have been translated into many common languages, the vast number of linguistically diverse verbal communities without access to these resources remain marginalized.

Systematic reviews of prior research tout many benefits of bilingual-language instruction for children with autism spectrum disorder (ASD; Lim et al., 2019; Wang et al., 2018). Other reviews point to specific foreign-language interventions, like tact training (Wooderson et al., 2022) and intraverbal training (Melvin-Brown et al., 2022), for evoking untrained operants within a second language. However, Petursdottir and Oliveira (2023) contend that the behavior-analytic literature on foreign-language acquisition remains meager.

Given the growing prevalence of multilingual speakers with ASD from culturally and linguistically diverse backgrounds, knowing how intervention focused on one language affects the acquisition of another would be beneficial. Moreover, incorporating native languages into intervention may improve treatment outcomes (Lim et al., 2019). Here we provide a case study of repeated functional analyses of verbal behavior for a multilingual boy with ASD over the course of ABA intervention and a language immersion experience with his family.

## Method

### Participant and Setting

Over the course of a year, we examined the verbal behavior of Ryan, a 7-year-old boy whose parents had emigrated from India. Ryan was exposed to multiple languages at home, including English, Telugu, and Tamil. At the start of the present study, Ryan’s verbal behavior consisted solely of single-word responses in English. Table 1 shows the languages used as a speaker and listener by members of Ryan’s verbal community.

**Table 1:** An overview of Ryan’s linguistically diverse verbal community

Speaker	Listener			
	Ryan	Mom	Dad	RBT
Ryan	—	English^a^	English^a^	English^a^
Mom	Telugu	—	Telugu	Tamil
Dad	English	Telugu	—	English/Tamil
RBT	English	Tamil	English/Tamil	—

Tamil and Telugu are two of India’s 22 official languages, primarily spoken in the southern part of the country. Tamil is spoken by people from the state of Tamil Nadu, and Telugu is spoken by those from the states of Andhra Pradesh and Telangana. Ryan’s parents spoke Telugu to one another. While his mother spoke to Ryan in Telugu, his father spoke to him in English. Both parents also spoke Tamil, though never to Ryan. The Registered Behavior Technician (RBT) assigned to Ryan’s case was also from India, and spoke English and Tamil, but not Telugu. Per the request of his parents, the RBT provided early intensive behavioral intervention to Ryan in English, but she primarily communicated with his parents in Tamil

^a^ One-word responses emitted in English. Ryan had never been heard to speak Telugu or Tamil

Several of the neighboring families were also from India, and Ryan’s parents primarily communicated with them in either Telugu or Tamil. Ryan’s multilingual verbal community presented a unique opportunity to assess his functional speech across non-English languages. This study took place in the participant’s home where he received early intensive behavioral intervention (EIBI) from a Registered Behavior Technician (RBT). A supervising Board Certified Behavior Analyst independently recorded the frequency and topography of Ryan’s tacts, mands, echoics, and intraverbals for all trials of every assessment. Trial-by-trial interobserver agreement measured 100%.

### Procedures

A verbal operant experimental (VOX) analysis (Enriquez et al., 2023) was conducted in English to assess Ryan’s present levels of functional speech. Since Skinner’s (1957) introduction of the elementary verbal operants, researchers studying language remediation in children with ASD have primarily focused on four sources of environmental control: mand, tact, intraverbal, and echoic (DeSouza et al., 2017). Given that pure sources of control rarely occur in the natural environment (Michael et al., 2011), we acknowledge the potential for supplementary variables.

A VOX analysis extends prior research on functional analyses of verbal behavior (see Plavnick & Normand, 2013) to examine the functional interdependence of verbal operants by assessing the same topographies under different sources of control. We plotted the frequency of verbal responses observed across each operant class on a radar chart to create a polygonal profile of Ryan’s verbal behavior. Polygonal language profiles allow for visual and quantitative analyses using a normalized first moment of area (*Q’*; Porter & Niksiar, 2018); a measure analogous to area under the curve, ranging from 0.00 to 2.00.

#### Initial Assessment

The VOX analysis began by allowing Ryan to select from a variety of preferred items to assess his *labeling* (i.e., tact control). Upon picking up an item, the RBT inquired, “What is it?” To control for potential tact confounds, the RBT ensured that: (a) access to the item was not restricted (i.e., mand control), (b) the name of the item was not provided (i.e., echoic control), nor (c) was a verbal description of the item presented (i.e., intraverbal control). If he labeled the item, the RBT provided praise. For example, while holding a gel-filled, thermoplastic rubber ball Ryan said, “Squishy,” and the RBT said, “Yes, it is a squishy!” After 20 s, the target item was removed, and Ryan was encouraged to select another response target. We repeated this process until we assessed labeling with three different items: “Pig,” “Squishy,” and “Turtle.”

Next, we used a multiple-stimulus without replacement preference assessment to assess Ryan’s *requesting* (i.e., mand control) for each of the three items identified in the labeling condition. We placed all three items on the floor in front of Ryan and asked him to choose one. Upon selecting one, the RBT removed the other two items. After 20 s, the RBT removed the target item and asked, “What do you want?” To control for potential mand confounds, the RBT ensured that: (a) the item was not physically present (i.e., tact control), (b) the name of the item was not provided (i.e., echoic control), nor (c) was a verbal description of the item presented (i.e., intraverbal control). If he requested the item, the RBT gave it to him. For example, having selected the squishy ball, the RBT hid it behind her back to evoke Ryan’s request, “Squishy.” When he said, “Squishy,” she gave it to him for another 20 s. The first item was removed, and Ryan was asked to select one of the two remaining items. We repeated this process repeated until we assessed requesting with all three items: “Squishy,” “Pig,” and “Turtle.”

Then we assessed Ryan’s *echoing* (i.e., echoic control)*,* using the name for each item emitted by Ryan in the prior conditions as antecedent verbal stimuli. Throughout these next two conditions, Ryan was engaged in an activity unrelated to the items being assessed to abolish their reinforcing value. To control for potential echoic confounds, the RBT ensured that: (a) the item was not physically present (i.e., tact control), (b) access to the item was not restricted (i.e., mand control), nor (c) was a verbal description of the item presented (i.e., intraverbal control). The three antecedent verbal stimuli were presented semi-randomly to systematically vary the order of presentation from the previous two conditions, thereby controlling for potential sequencing effects. For each word he echoed, the RBT provided praise. For example, the RBT said, “Say, squishy.” If Ryan said “Squishy,” the RBT replied, “Good saying squishy!” We continued this process until we assessed echoing the names of all three items: “Turtle,” “Pig,” and “Squishy.”

Finally, we assessed Ryan’s *conversing* (i.e., intraverbal control) about each of the three items. Ryan was engaged in an activity unrelated to any of the response targets, and at 20-s intervals, he was asked to complete a fill-in-the-blank statement specific to Ryan’s interaction with each item. These intraverbal frames were structured to occasion Ryan’s response to complete the sentence by saying the name of a corresponding response target. To control for potential intraverbal confounds, the RBT ensured that: (a) the item was not physically present (i.e., tact control), (b) access to the item was not restricted (i.e., mand control), nor (c) was the name of the item provided (i.e., echoic control). Additionally, the order in which the RBT presented the three fill-ins was varied intentionally from the previous three conditions to control for potential sequencing effects. If Ryan answered the fill-ins, the RBT provided praise. For example, while Ryan was playing with a musical toy, the RBT said, “You squeeze the ….” If Ryan’s said, “Squishy,” the RBT enthusiastically repeated, “Squishy!” We repeated this process until we assessed conversing about all three items: “Pig,” “Turtle,” and “Squishy.”

After Ryan’s functional language had been assessed for each of the three items, a second round of the assessment was conducted. The Labeling condition came first to allow Ryan to select three novel verbal response targets, but we varied the sequence of the other conditions from the first round: Conversing, Echoing, and Requesting. Two rounds of each condition were necessary to achieve an adequate sample size to analyze Ryan’s verbal behavior.^1^ All conditions were completed in one session, with the total assessment lasting approximately 30 min.

#### 6-month Reassessment

Another VOX analysis was conducted to assess progress and identify present levels of functional language. Since the initial VOX analysis, 30-h per week of EIBI had been conducted in English. Mom continued to speak to Ryan in Telugu, and Dad spoke to him in English. Ryan – now 7.5 years old – continued to be exposed to Tamil intermittently, but we did not observe his verbal community directly reinforcing Tamil as either speaker or listener. We reconducted the VOX analysis as described above.

To comprehensively assess Ryan’s verbal behavior, we conducted a VOX in Telugu and another in Tamil. The procedures in both assessments were identical to those used in the English assessment except that the RBT delivered antecedent verbal stimuli in Telugu or Tamil and consequences followed vocalizations in Telugu or Tamil.^2^ That is, for the Labeling conditions, the RBT called for Ryan to respond in English (e.g., “What is that?”), Telugu (e.g., “Adi ēmiṭi?”), or Tamil (e.g., “Atu eṉṉa?”). For the Requesting conditions, the RBT called for Ryan to respond in English (e.g., “What do you want?”), Telugu (e.g., “Nīku ēmi kāvāli?”), or Tamil (e.g., “Uṅakku eṉṉa vēṇnum?”). For the Echoing conditions, the RBT called for Ryan to respond in English (e.g., “Say …”), Telugu (e.g., “Ceppu …”), or Tamil (e.g., “Sollu …”). For the Conversing conditions, the RBT called for Ryan to respond in English (e.g., “Choo, choo goes the …”), Telugu (e.g., “Cū, cū veḷtāḍu …”), or Tamil (e.g., “Cū, ccū celkiṟatu …”).

#### 12-month Reassessment

The intensity of Ryan’s behavior-analytic intervention was titrated down for the subsequent 6-month treatment authorization. Ryan received only six hours per week of focused behavior-analytic intervention, and he attended school two days per week. Furthermore, his services were interrupted for consecutive weeks when COVID-19 spread through his family, and again when his family traveled to a Tamil-speaking region of India for a month. At the end of the second 6-month authorization period, Ryan was 8 years old. We conducted three VOX analyses in English, Telugu, and Tamil to assess Ryan’s functional verbal behavior across languages.

## Results and Discussion

**Fig. 1:**
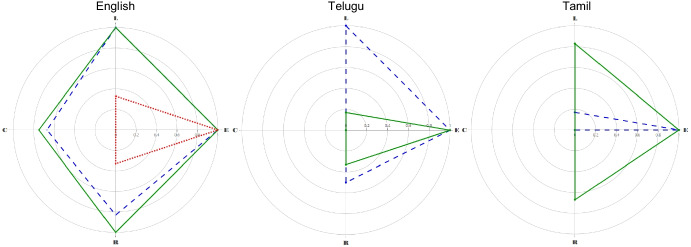
Radar charts for Ryan’s VOX analyses across three languages. *Note:* First moment of area (Q’) was used to measure the size of Ryan’s speaking repertoire at baseline (dotted), 6-months (dashed), and 12-months (solid) across the three languages of his verbal community*.* Clockwise from left on each radar chart: C = Conversing, L = Labeling, E = Echoing, and R = Requesting

Figure 1 displays the results of each VOX analysis conducted with Ryan. The three English-language VOX analyses are shown on the far left chart. During the initial assessment, Ryan did not converse about any of the items within the assessment, but he demonstrated echoing (100%), labeling (33%), and requesting (33%). The size of Ryan’s initial English-language profile was calculated at *Q’* = 0.22 (*A* = 0.33, *R* = 0.33).

At his 6-month reassessment, Ryan demonstrated conversing (67%), labeling (100%), echoing (100%), and requesting (83%). Accordingly, his English-language profile increased markedly to *Q’* = 1.34 (A = 1.53, R = 0.12). The 12-month reassessment showed that his English-language profile continued to expand across conversing (75%), labeling (100%), echoing (100%), and requesting (100%): *Q’* = 1.60 (A = 1.75, R = 0.08).

The two Telugu-language VOX analyses are displayed in the middle chart. After 6 months of English-language EIBI, Ryan demonstrated labeling (100%), echoing (100%), and requesting (50%) in Telugu: *Q’* = 0.47 (*A* = 0.75, *R* = 0.37). These results were not sustained at his 12-month reassessment, however, which showed labeling (17%), echoing (100%), and requesting (33%). Ryan’s Telugu-language profile had diminished to *Q’* = 0.17 (*A* = 0.25, *R* = 0.34). Conversing (0%) was absent from both Telugu assessments.

The two Tamil-language VOX analyses are shown on the far right chart. After 6 months of English-language EIBI, Ryan demonstrated labeling (17%) and echoing (100%) in Tamil, but neither conversing (0%) nor requesting (0%): *Q’* = 0.06 (*A* = 0.08, *R* = 0.34). At his 12-month reassessment, Ryan’s labeling (83%), echoing (100%), and requesting (67%) increased his Tamil-language profile to *Q’* = 0.50 (*A* = 0.75, *R* = 0.34). Conversing (0%) remained absent.

At the time of his 6-month reassessment, Ryan’s behavior as a speaker and listener of English had been explicitly reinforced, and his behavior as a listener – but not as a speaker – of Telugu had been similarly reinforced. He had been exposed to Tamil, but it had never been conditioned as either speaker or listener behavior. Since neither of Ryan’s parents ever spoke to him in Tamil, they reported their surprise to hear him speak it.

Ryan demonstrated strong echoing across all languages. The recombinative minimal unit of the echoic repertoire likely facilitated Ryan’s echoing in Telugu and Tamil. Consequently, Ryan may have also echoed antecedent verbal stimuli from other languages, beyond those spoken by his own verbal community. It is unlikely, however, that Ryan would converse, label, or request in a novel language without the support of a verbal community.

The reversal in size of Ryan’s Telugu and Tamil repertoires between the two reassessments corresponds with his month-long immersion in a Tamil-speaking region of India. The tenuous nature of Ryan’s Telugu- and Tamil-speaking repertoires highlights important areas of future research. His English conversation skills likely contributed to the maintenance of Ryan’s English-speaking repertoire throughout his immersive experience in India. Conversing was never established in Telugu or Tamil, which may explain their susceptibility to extinction (Bouton et al., 2021; Melvin-Brown et al., 2022). Future research should continue to explore the role of intraverbal control on the maintenance of a diverse verbal repertoire.

A primary limitation to the current study is the omission of baseline levels of Telugu and Tamil, which afforded only a quasi-experimental analysis of Ryan’s heritage language skills. At the time of the initial assessment, there was no treatment-specific reason to assess his heritage language skills because Ryan’s English was so severely limited. Although Ryan’s parents reported that they had never heard him speak either Telugu or Tamil, we cannot rule out the possibility that an operant level of these languages already existed. Additionally, no procedural fidelity data are available. We urge other behavior analysts to assess the functions of all languages to which a speaker is exposed within their verbal community or communities.

This research was born of the context in which a child with ASD was immersed in a multilingual verbal community. The longitudinal nature of the current study could only be achieved by sacrificing some degree of experimental control. Nevertheless, it is clear that the acquisition of a functional verbal repertoire across languages is contingent on the reinforcement of the verbal community. The changing dynamics of American society will continue to provide opportunities to analyze the verbal behavior of individuals from culturally and linguistically diverse backgrounds, and behavior analysts are uniquely situated to follow Skinner’s (1956) advice to drop everything else and study it.

## Data Availability

All data generated or analyzed during this study are included in the published article.

## Footnotes

1 Up to four rounds of assessment may be required. See Mason et al. (2022) for an extended discussion of statistical power for a VOX analysis.

2 All assessments were completed on the same day, with a half-hour break between languages. The RBT spoke Tamil and understood Telugu. Ryan’s mother prompted the RBT how to call for responses in Telugu.
